# Baseline Methods for Bayesian Inference in Gumbel Distribution

**DOI:** 10.3390/e22111267

**Published:** 2020-11-07

**Authors:** Jacinto Martín, María Isabel Parra, Mario Martínez Pizarro, Eva L. Sanjuán

**Affiliations:** 1 Departamento de Matemáticas, Facultad de Ciencias, Universidad de Extremadura, 06006 Badajoz, Spain; jrmartin@unex.es (J.M.); mipa@unex.es (M.I.P.); 2 Departamento de Matemáticas, Facultad de Veterinaria, Universidad de Extremadura, 10003 Cáceres, Spain; mariomp@unex.es; 3 Departamento de Matemáticas, Centro Universitario de Mérida, Universidad de Extremadura, 06800 Mérida, Spain

**Keywords:** Bayesian inference, highly informative prior, Gumbel distribution, small dataset

## Abstract

Usual estimation methods for the parameters of extreme value distributions only employ a small part of the observation values. When block maxima values are considered, many data are discarded, and therefore a lot of information is wasted. We develop a model to seize the whole data available in an extreme value framework. The key is to take advantage of the existing relation between the baseline parameters and the parameters of the block maxima distribution. We propose two methods to perform Bayesian estimation. Baseline distribution method (BDM) consists in computing estimations for the baseline parameters with all the data, and then making a transformation to compute estimations for the block maxima parameters. Improved baseline method (IBDM) is a refinement of the initial idea, with the aim of assigning more importance to the block maxima data than to the baseline values, performed by applying BDM to develop an improved prior distribution. We compare empirically these new methods with the Standard Bayesian analysis with non-informative prior, considering three baseline distributions that lead to a Gumbel extreme distribution, namely Gumbel, Exponential and Normal, by a broad simulation study.

## 1. Introduction

Extreme value theory (EVT) is a widely used statistical tool for modeling and forecasting the distributions which arise when we study events that are more extreme than any previously observed. Examples of these situations are natural rare events in climatology or hydrology, such as floods, earthquakes, climate changes, etc. Therefore, EVT is employed in several scientific fields, to model and predict extreme events of temperature [1,2,3], precipitation [4,5,6,7,8,9,10] and solar climatology [11,12,13], as well as in the engineering industry to study important malfunctions (e.g., [14], finance (study of financial crisis), insurance (for very large claims due to catastrophic events) and environmental science (concentration of pollution in the air).

Overall fitting method tries to fit all the historical data to several theoretical distributions and then choose the best one, according to certain criteria. However, since the number of extreme observations is usually scarce, overall fitting works well in the central distribution area, but it can poorly represent the tail area.

In EVT, there are two main approaches, block maxima (BM) method and peak-over-threshold (POT), which are differentiated by the way each model classifies the observations considered as extreme events and then uses them in the data analysis process. In the POT method, the extreme data are the ones over a certain threshold, while, for BM method, data are divided into blocks of equal size, and the maximum datum for each block is selected. BM method is preferable to POT when the only available information is block data, when seasonal periodicity is given or the block periods appear naturally, such as in studies for temperature, precipitation and solar climatology. The biggest challenge in BM method is deciding the size of the blocks when they are not obvious.

The Gumbel distribution plays an important role in extreme value analysis as model for block maxima, because it is appropriate to describe extreme events from distributions such as Normal, Exponential and Gumbel distributions [15]. To estimate maximum data distribution, both frequentist and Bayesian approaches have been developed [16,17]. However, the knowledge of physical constraints, the historical evidence of data behavior or previous assessments might be an extremely important matter for the adjustment of the data, particularly when they are not completely representative and further information is required. This fact leads to the use of Bayesian inference to address the extreme value estimation [18].

Practical use of Bayesian estimation is often associated with difficulties to choose prior information and prior distribution for the parameters of the extreme values distribution [19]. To fix this problem, several alternatives have been proposed, either by focusing exclusively on the selection of the prior density [20,21] or by improving the algorithm for the estimation of the parameters [22]. However, the lack of information still seems to be the weakness when referring to extreme value inference.

Examples for its application are the modeling of annual rainfall maximum intensities [23], the estimation of the probability of exceedence of future flood discharge [24] and the forecasting of the extremes of the price distribution [25]. Some of these works are focused on the construction of informative priors of the parameters for which data can provide little information. Despite these previous efforts, it is well understood that some constraints to quantify qualitative knowledge always appear when referring to construct informative priors.

Therefore, this paper focuses on techniques to employ all the available data in order to elicit a highly informative prior distribution. We consider several distributions that lead to a Gumbel extreme distribution. The key is to take advantage of the existing relation between the baseline parameters and the parameters of the block maxima distribution. The use of the entire dataset, instead of the selected block maximum data, results to be adequate and it is advisable when dealing with very shortened available data.

We employ MCMC techniques, concretely a Metropolis–Hastings algorithm. Several statistical analyses are performed to test the validity of our method and check its enhancements in relation to the standard Bayesian analysis without this information.

## 2. Domains of Attraction of Gumbel Distribution

As is well known, the BM approach consists on dividing the observation period into non overlapping periods of equal size and select the maximum observation in each period. Given a sequence of i.i.d. random variables $Y_{1},Y_{2},\ldots ,Y_{m}$ with common distribution function *F*, and given a fixed k∈N (block size), we define the block maxima

(1) $$X_{i}=\max_{(i-1)k<j\leq ik}Y_{j},\;i=1,2,\ldots ,n.$$

Hence, the total observations, m=k×n, are divided into *n* blocks of size *k*. The extreme values depend upon the full sample space from which they have been drawn through its shape and size. Therefore, extremes variate according to the initial distribution and sample size ([26]). Then, the cumulative distribution function of $X_{i}$ is

(2) $$P\left(X_{i}\leq x\right)=P\left(Y_{(i-1)k+1}\leq x,\ldots ,Y_{ik}\leq x\right)=\Pi_{j=(i-1)k+1}^{ik}P\left(Y_{j}\leq x\right)=F{\left(x\right)}^{k}$$

This result depends on our knowledge of *F*, in which we could be lacking. Therefore, it is useful to consider the asymptotic distribution.

According to the Gnedenko [27] and Fisher and Tippett [28] theorems, the asymptotic distribution of block maxima of random i.i.d. variables can be approximated by a generalized extreme value distribution, with distribution function

(3) $$GEV\left(x;\xi ,\mu ,\sigma \right)=\exp\left\{-{\left(1+\xi \left(\frac{x-\mu }{\sigma }\right)\right)}^{-1/\xi }\right\}$$

with ξ,μ∈R,σ>0 and $1+\xi \left(\frac{x-\mu }{\sigma }\right)>0$.

When ξ=0, the right-hand side of Equation (3) is interpreted as

(4) $$G\left(x;\mu ,\sigma \right)=\exp\left\{-\exp\left\{-\left(\frac{x-\mu }{\sigma }\right)\right\}\right\}$$

and it is called Gumbel distribution with parameters μ (location) and σ>0 (scale).

**Definition** **1.** *We say that the distribution function F is in the domain of attraction of a extreme value Gumbel distribution when there exist sequences $\left\{a_{k}\right\}$ and $\left\{b_{k}\right\}$, with $a_{k}>0,b_{k}\in R$ such that*

(5) $$\lim_{k\to \infty }F^{k}\left(a_{k}x+b_{k}\right)\;=\;G\left(x\right),\;x\in R$$

Sequences $\left\{a_{k}\right\}$ and $\left\{b_{k}\right\}$ are called normalizing constants. We usually call the distribution *F* baseline or underlying distribution. Normalizing constants correspond to the parameters of scale and location of the limit Gumbel distribution, therefore they allow us to establish a relation between this distribution and the baseline distribution.

Moreover, Ferreira and de Haan [29] showed theoretical results which allow determining the normalizing constants for many baseline distributions in the domain of attraction of a Gumbel distribution:

**Theorem** **1.** *When F belongs to the domain of attraction of a Gumbel distribution, there is a positive function h that verifies*

(6) $$a_{k}=h\left(b_{k}\right),\;b_{k}=F^{-1}\left(1-k^{-1}\right),\;\forall k.$$

To determine function *h*, the following condition is very useful.

**Theorem** **2** (Von-Mises condition)**.** When $F^{\prime \prime }\left(x\right)$ and $F^{\prime }\left(x\right)$ exist, and $F^{\prime }$ is positive for all x belonging to a neighborhood at the left of $x^{*}$ (right endpoint of F), and

(7) $$\lim_{t\to x^{*}}{\left(\frac{1-F}{F^{\prime }}\right)}^{\prime }\left(t\right)=0,$$

*or equivalently*

(8) $$\lim_{t\to x^{*}}\frac{\left(1-F(t)\right)\cdot F^{\prime \prime }\left(t\right)}{{\left(F^{\prime }\left(t\right)\right)}^{2}}=-1,$$

*then F belongs to the domain of attraction of the Gumbel distribution. In this case, function h is determined by*

(9) $$h\left(t\right)=\frac{1-F(t)}{F^{\prime }\left(t\right)}$$

Distributions whose tails decrease exponentially produce a Gumbel distribution when taking the block maxima. Besides the Exponential distribution, the class of distributions which belong to the domain of attraction of the Gumbel includes the Normal distribution, and many others, such as Log-normal, Gamma, Rayleigh, Gumbel, etc.

We also use the following result:

**Proposition** **1.** *If X is a random variable belonging to the domain of attraction of a Gumbel distribution, then Y=μ+σX also belongs to the same domain of attraction. The normalization constants are:*

(10) $${\tilde{a}}_{k}=\sigma a_{k},\;\;\;{\tilde{b}}_{k}=\mu +\sigma b_{k}.$$

*where $a_{k}$ and $b_{k}$ are the normalization constants of X.*

### 2.1. Gumbel Baseline Distribution

If $Y∼G\left(\mu ,\sigma \right)$, then block maxima distribution of size *k*, denoted by *X*, is also a Gumbel distribution, because

(11) $$F{\left(x\right)}^{k}=\exp\left\{-\exp\left(-\frac{x-\mu }{\sigma }\right)+k\right\}=\exp\left\{-\exp\left(-\frac{x-\left(\mu +\sigma \ln k\right)}{\sigma }\right)\right\},$$

therefore $X∼G\left(\mu +\sigma \ln k,\sigma \right)$.

### 2.2. Exponential Baseline Distribution

Let $Y∼Exp\left(\lambda \right)$ with distribution function

(12) $$F\left(y\right)=1-e^{-\lambda y},\;\;\;y\;\geq 0,$$

Exponential distribution belongs to the domain of attraction of the Gumbel, with $h\left(t\right)=\lambda^{-1}$. As $F^{-1}\left(u\right)=\lambda^{-1}\ln\left(1-u\right)$, the normalization constants are

(13) $$a_{k}=\lambda^{-1},\;\;\;\;b_{k}=\lambda^{-1}\;\ln k,$$

and they settle a relation that allow us to make estimations for Gumbel limit distribution, when there is an exponential baseline distribution for *k* big enough.

### 2.3. Normal Baseline Distribution

When the baseline distribution is a Standard Normal distribution, normalizing constants can be computed, making use of asymptotic limit and results showed before.

Let Z∼N(0,1), with distribution function *F* and density function *f*. It is easy to show that *F* verifies von Mises condition (8):

$$\begin{array}{ccc} \lim_{t\to x^{*}}\frac{\left(1-F(t)\right)\cdot F^{\prime \prime }\left(t\right)}{{\left(F^{\prime }\left(t\right)\right)}^{2}} & = & \lim_{t\to x^{*}}\frac{-\left(1-F(t)\right)\cdot f\left(t\right)t}{{\left(f(t)\right)}^{2}}=\lim_{t\to x^{*}}\frac{-\left(1-F(t)\right)\cdot t}{f(t)}\\ & = & \lim_{t\to x^{*}}\frac{-\left(1-F(t)\right)+f\left(t\right)\cdot t}{f^{\prime }\left(t\right)}=\lim_{t\to x^{*}}\frac{f(t)\cdot t}{-t\cdot f(t)}=-1 \end{array}$$

using L’Hôpital and noticing that $f^{\prime }\left(t\right)=-t\cdot f\left(t\right)$. Therefore, $1-F\left(t\right)\approx f\left(t\right)\cdot t^{-1}$, and, consequently, the function *h* defined as (9) verifies

$$\lim_{t\to x^{*}}h\left(t\right)=t^{-1}$$

Besides, by (6), $F\left(b_{k}\right)=1-k^{-1}$. Therefore, $\ln\left(1-F(b_{k})\right)=-\ln k$, or

$$\ln f\left(b_{k}\right)-\ln b_{k}=-\ln k,$$

so

(14) $$b_{k}^{2}+\ln\left(2\pi \right)+2\ln b_{k}=2\ln k.$$

Defining the function $g\left(b_{k}\right)=b_{k}^{2}+\ln\left(2\pi \right)+2\ln b_{k}-2\ln k$, and developing its Taylor series around ${\left(2\ln k\right)}^{1/2}$, we obtain

$$\begin{array}{cc} g\left(b_{k}\right) & =g\left({\left(2\ln k\right)}^{1/2}\right)+g^{\prime }\left({\left(2\ln k\right)}^{1/2}\right)\cdot \left(b_{k}-{\left(2\ln k\right)}^{1/2}\right)+O\left({\left(2\ln k\right)}^{1/2}\right)\\ & =\left[\ln\ln k+\ln4\pi \right]+2\left[{\left(2\ln k\right)}^{1/2}+{\left(2\ln k\right)}^{-1/2}\right]\cdot \left(b_{k}-{\left(2\ln k\right)}^{1/2}\right)\\ & +O\left({\left(2\ln k\right)}^{1/2}\right), \end{array}$$

so, as $g(b_{k})=0$, for *k* big enough

(15) $$b_{k}={\left(2\ln k\right)}^{1/2}-2^{-1}{\left(2\ln k\right)}^{-1/2}\left[\ln\left(\ln k\right)+\ln\left(4\pi \right)\right].$$

In addition, as $a_{k}=h\left(b_{k}\right)\approx b_{k}^{-1}$ and

(16) $$b_{k}^{-1}=\frac{1}{{\left(2\ln k\right)}^{1/2}-2^{-1}{\left(2\ln k\right)}^{-1/2}\left[\ln\left(\ln k\right)+\ln\left(4\pi \right)\right]}\approx {\left(2\ln k\right)}^{-1/2}$$

$a_{k}$ can be taken as

(17) $$a_{k}\approx {\left(2\ln k\right)}^{-1/2}.$$

Besides, as a consequence of Theorem 10, if $Y∼N\left(\mu ,\sigma \right)$, for *k* big enough, the normalization constants are, approximately,

(18) $$a_{k}=\sigma {\left(2\ln k\right)}^{-1/2},\;\;b_{k}=\mu +\sigma \left[{\left(2\ln k\right)}^{1/2}-2^{-1}{\left(2\ln k\right)}^{-1/2}\left[\ln\ln k+\ln\left(4\pi \right)\right]\right].$$

### 2.4. Other Baseline Distributions

This way of working can be extended to other baseline distributions, whose block maxima limit is also a Gumbel, by using existing relations between baseline and limit parameters. In Table 1, normalization constants computed for the most employed distribution functions in the domain of attraction of the Gumbel distribution are shown. Constants $a_{N}$ and $b_{N}$ are the normalization constants for Standard Normal distribution, given by (15) and (17), respectively.

## 3. Bayesian Estimation Methods

### 3.1. Classical Bayesian Estimation for the Gumbel Distribution

To make statistical inferences based on the Bayesian framework, after assuming a prior density for the parameters, π(θ), and combining this distribution with the information brought by the data which are quantified by the likelihood function, L(θ|x), the posterior density function of the parameters can be determined as

(19) π(θ|x)∝L(θ|x)π(θ)

The remaining of the inference process is fulfilled based on the obtained posterior distribution.

The likelihood function for θ=(μ,σ), given the random sample $x=(x_{1},\ldots ,x_{n})$ from a Gumbel(μ,σ) distribution, with density function given by

(20) $$f\left(x|\mu ,\sigma \right)=\frac{1}{\sigma }\exp\left(-\exp\left(-\frac{x-\mu }{\sigma }\right)-\frac{x-\mu }{\sigma }\right)$$

where μ∈R, $\sigma \in R_{+}^{*}$, is

(21) $$L\left(\mu ,\sigma |x\right)=\frac{1}{\sigma^{n}}\exp\left(\Delta \right),$$

with

(22) $$\Delta =-\sum_{i=1}^{n}\exp\left(-\frac{x_{i}-\mu }{\sigma }\right)-\sum_{i=1}^{n}\frac{x_{i}-\mu }{\sigma }.$$

In the case of the Gumbel distribution, Rostami and Adam [21] selected eighteen pairs of priors based on the parameters’ characteristics, assumed independence, and compared the posterior estimations by applying Metropolis–Hastings (MH) algorithm, concluding that the combination of Gumbel and Rayleigh is the most productive pair of priors for this model. For fixed initial hyper-parameters $\mu_{0},\sigma_{0},\lambda_{0}$

(23) $$\begin{array}{cc} \pi (\mu ) & \propto \exp\left(-\exp\left(-\frac{\mu -\mu_{0}}{\sigma_{0}}\right)-\frac{\mu -\mu_{0}}{\sigma_{0}}\right)\\ \pi (\sigma ) & \propto \sigma \exp\left(-\frac{\sigma^{2}}{2\lambda_{0}^{2}}\right). \end{array}$$

The posterior distribution is

(24) $$\pi \left(\mu ,\sigma |x\right)\propto \frac{1}{\sigma^{n-1}}\exp\left(\Delta -\exp\left(-\frac{\mu -\mu_{0}}{\sigma_{0}}\right)-\frac{\mu -\mu_{0}}{\sigma_{0}}-\frac{\sigma^{2}}{2\lambda_{0}^{2}}\right),$$

and conditional posterior distributions are

(25) $$\begin{array}{ccc} \pi (\mu |\sigma ,x) & \propto & \exp\left(\Delta -\exp\left(-\frac{\mu -\mu_{0}}{\sigma_{0}}\right)-\frac{\mu -\mu_{0}}{\sigma_{0}}\right)\\ \pi (\sigma |\mu ,x) & \propto & \frac{1}{\sigma^{n-1}}\exp\left(\Delta -\frac{\sigma^{2}}{2\lambda_{0}^{2}}\right) \end{array}$$

Then, an MCMC method is applied through the MH algorithm.

1. Draw a starting sample $\left(\mu^{\left(0\right)},\sigma^{\left(0\right)}\right)$ from starting distributions, π(μ),π(σ), respectively, given by Equation (23).
2. For j=0,1,…, given the chain is currently at $\mu^{\left(j\right)},\sigma^{\left(j\right)}$,
  - Sample candidates $\mu^{*},\sigma^{*}$ for the next sample from a proposal distribution,
    $$\mu^{*}∼N\left(\mu^{\left(j\right)},v_{\mu }\right)\;\mathrm{and}\;\sigma^{*}∼N\left(\sigma^{\left(j\right)},v_{\sigma }\right)$$
  - Calculate the ratios
    (26) $$r_{\mu }=\frac{\pi (\mu^{*}|\sigma^{\left(j\right)},x)}{\pi (\mu^{\left(j\right)}|\sigma^{\left(j\right)},x)},\;r_{\sigma }=\frac{\pi (\sigma^{*}|\mu^{\left(j\right)},x)}{\pi (\sigma^{\left(j\right)}|\mu^{\left(j\right)},x)}$$
  - Set
    (27) $$\mu^{\left(j+1\right)}=\left\{\begin{array}{cc} \mu^{*}, & \mathrm{with}\;\mathrm{probability}\min\{1,r_{\mu }\}\\ \mu^{\left(j\right)}, & \mathrm{otherwise} \end{array}\right.$$
    (28) $$\sigma^{\left(j+1\right)}=\left\{\begin{array}{cc} \sigma^{*}, & \mathrm{with}\;\mathrm{probability}\min\{1,r_{\sigma }\}\\ \sigma^{\left(j\right)}, & \mathrm{otherwise} \end{array}\right.$$
3. Iterate the former procedure. Notice that
  $$\begin{array}{c} \begin{array}{cc} r_{\mu } & =\exp\left\{\frac{n}{\sigma^{\left(j\right)}}\left(\mu^{*}-\mu^{j}\right)+\frac{\mu^{\left(j\right)}-\mu^{*}}{\sigma_{0}}+\exp\left(-\frac{\mu^{\left(j\right)}-\mu_{0}}{\sigma_{0}}\right)-\exp\left(-\frac{\mu^{*}-\mu_{0}}{\sigma_{0}}\right)\right.\\ & \left.+\sum_{i=1}^{n}\left[\exp\left(-\frac{x_{i}-\mu^{\left(j\right)}}{\sigma^{\left(j\right)}}\right)-\exp\left(-\frac{x_{i}-\mu^{*}}{\sigma^{\left(j\right)}}\right)\right]\right\}\\ r_{\sigma } & ={\left(\frac{\sigma^{\left(j\right)}}{\sigma^{*}}\right)}^{n-1}\exp\left\{\frac{{\left(\sigma^{\left(j\right)}\right)}^{2}-{\left(\sigma^{*}\right)}^{2}}{2\lambda_{0}^{2}}+\left(\frac{1}{\sigma^{\left(j\right)}}-\frac{1}{\sigma^{*}}\right)\sum_{i=1}^{n}\left(x_{i}-\mu^{\left(j\right)}\right)\right.\\ & \left.+\sum_{i=1}^{n}\left[\exp\left(-\frac{x_{i}-\mu^{\left(j\right)}}{\sigma^{\left(j\right)}}\right)-\exp\left(-\frac{x_{i}-\mu^{\left(j\right)}}{\sigma^{*}}\right)\right]\right\}. \end{array} \end{array}$$

Therefore, we obtain a Markov chain that converges to the posterior distributions for the parameters μ and σ. We call this method Classical Metropolis–Hastings method (MHM).

### 3.2. Baseline Distribution Method

In Baseline distribution method (BDM), we take all the information available to determine posterior baseline distribution. We denote B(θ) as the baseline distribution with parameter vector θ.

Then, we can apply Bayesian inference procedures to estimate the posterior distribution of the baseline distribution, denoted by $\pi_{b}\left(\theta |y\right)$ and, therefore, to obtain estimations for the parameters of the baseline distribution θ, with all the data provided by y.

Afterwards, making the transformation given by the relations we obtained in previous section, we can obtain new estimations for the parameters of block maxima distribution, which is the Gumbel in this case. We explain the procedure for the three baseline distributions considered in this paper: Gumbel, Exponential and Normal distribution.

#### 3.2.1. Gumbel Baseline Distribution

When the baseline distribution $Y∼G\left(\mu_{b},\sigma_{b}\right)$, it is known that the limit distribution $X∼G\left(\mu_{b}+\sigma_{b}\ln k,\sigma_{b}\right)$. Therefore, MH algorithm can be applied to the whole dataset, y, to find estimations for $\mu_{b}$ and $\sigma_{b}$. Afterwards, we make the adequate transformation to compute estimations for the parameters of *X*.

#### 3.2.2. Exponential Baseline Distribution

When the baseline distribution $Y∼Exp\left(\lambda_{b}\right)$, we consider a Gamma distribution with parameters $\alpha_{0}$ and $\beta_{0}$ as prior distribution

(29) $$\pi \left(\lambda_{b}\right)\propto \lambda_{b}^{\alpha_{0}-1}\exp\left(-\beta_{0}\lambda_{b}\right).$$

Therefore, the posterior distribution is

(30) $$\pi \left(\lambda_{b}|y\right)∼\Gamma \left(\alpha_{0}+m,\beta_{0}+\sum_{j=1}^{m}y_{j}\right),$$

thus Gibbs algorithm can be employed to generate samples of posterior distribution $\pi \left(\lambda_{b}|y\right)$. Once the estimation of $\lambda_{b}$ is obtained, *k*th power of the distribution function will be the estimation for block maxima distribution function of size *k*.

#### 3.2.3. Normal Baseline Distribution

Finally, when the baseline distribution $Y∼N\left(\mu_{b},\sigma_{b}\right)$, we employ Normal and inverse Gamma prior distributions

(31) $$\begin{array}{c} \pi \left(\;\mu_{b}\;\right)\propto \exp\left(-\frac{\sigma_{0}}{2\sigma_{b}^{2}}{\left(\mu -\mu_{0}\right)}^{2}\right),\\ \pi \left(\;\sigma_{b}^{2}\;\right)\propto {\left(\frac{1}{\sigma_{b}^{2}}\right)}^{\alpha_{0}-1}\exp\left(-\frac{\beta_{0}}{\sigma_{b}^{2}}\right). \end{array}$$

Therefore, posterior distributions are

(32) $$\begin{array}{c} \pi \left(\;\mu_{b}|y,\sigma_{b}^{2}\;\right)∼N\left(\frac{\sigma_{0}\mu_{0}+\sum_{j=1}^{m}y_{j}}{\sigma_{0}+m},\;\;\frac{\sigma_{b}^{2}}{\sigma_{0}+m}\right),\\ \pi \left(\;\sigma_{b}^{2}|y,\mu_{b}\;\right)∼\mathrm{Inv}\Gamma \left(\frac{m}{2}+\alpha_{0},\;\beta_{0}+\frac{1}{2}\sum_{j=1}^{m}{\left(y_{j}-\mu_{b}\right)}^{2}\right), \end{array}$$

and we can employ Gibbs algorithm to generate samples of posterior distribution, and, afterwards, the *k*th power of the distribution function, as in the previous case.

### 3.3. Improved Baseline Distribution Method

Finally, we propose a new method, called Improved Baseline distribution method (IBDM), to import the highly informative baseline parameters into the Bayesian inference procedure. Here, we take into account the spirit of classical EVT, which grants more weight to block maxima data than to baseline data.

The method consists on applying BDM to obtain the posterior distribution for the parameters of the baseline distribution π(θ|y), and then uses it to build a prior distribution for the parameters of the Gumbel. Therefore, we have a highly informative prior distribution.

As the priors are highly informative, $\pi \left(\theta^{*}\right)=\pi \left(\theta^{\left(j\right)}\right)$, the ratio in the *j*th step of MH algorithm is

$$\begin{array}{cc} r_{\theta }=\frac{L\left(\mu^{*},\sigma^{*}|x\right)}{L\left(\;\mu^{\left(j\right)},\sigma^{\left(j\right)}|x\right)} & ={\left(\frac{\sigma^{\left(j\right)}}{\sigma^{*}}\right)}^{n}\exp\left\{\sum_{i=1}^{n}\left[\exp\left(-\frac{x_{i}-\mu^{\left(j\right)}}{\sigma^{\left(j\right)}}\right)\right.\right.\\ & \left.\left.-\exp\left(-\frac{x_{i}-\mu^{*}}{\sigma^{*}}\right)+\frac{x_{i}-\mu^{\left(j\right)}}{\sigma^{\left(j\right)}}-\frac{x_{i}-\mu^{*}}{\sigma^{*}}\right]\right\}. \end{array}$$

For every iteration of the algorithm, we first make an iteration of Baseline Distribution method, resulting $\theta_{b}$ as estimation of the posterior distribution $\pi (\theta_{b}|y)$. Afterwards, a candidate $\theta^{*}$ is generated using a Normal distribution $N(f\left(\theta_{b}\right),\nu_{\theta })$ with the adequate transformation $f(\theta_{b})$, given by Equations (11), (13) and (18) in the case of Gumbel, Exponential or Normal baseline distributions, respectively.

Obviously, this method is quite similar to BDM when block size is big and, consequently, there are few maxima data. It approaches the classical Bayesian method as the block size gets smaller (more maxima data).

## 4. Simulation Study

We made a simulation study for the three distributions analyzed above, which belong to the domain of attraction of the Gumbel distribution: Gumbel, Exponential and Normal.

For each distribution selected (once its parameters are fixed), we generated $m_{ij}=n_{i}\times k_{j}$ values, where

- $n_{i}$ is the number of block maxima, $n_{i}=2^{i},i=1,2\ldots ,7$; and
- $k_{j}$ is the block size, $k_{j}=10^{j},j=1,2,3.$

Therefore, the sample sizes vary from 20 to 128,000. Besides, each sequence is replicated 100 times. Consequently, 2100 sequences of random values were generated for each combination of parameters of each baseline distribution.

To guarantee the convergence of the MCMC algorithm, we must be sure that the posterior distribution has been reached. Some proceedings are advisable to be performed.

- Burn-in period: Eliminate the first generated values.
- Take different initial values and select them for each sample.
- Make a thinning to assure lack of autocorrelation.

These proceedings were made using library coda [30] for R software, taking 3000 values for the burn-in period, 50 values for the thinning and selecting initial values for each sample. Finally, to get the posterior distribution for each parameter, a Markov chain of length 10,000 was obtained. Therefore, 53,000 iterations were made for each sequence.

There are some common features for the baseline distributions considered when comparing the three methods MHM, BDM and IBDM.

1. To choose an estimator for the parameters, we compared mean- and median-based estimations. They were reasonably similar, due to the high symmetry of posterior distributions. Therefore, we chose the mean of the posterior distribution to make estimations of the parameters.
2. MHM usually provides high skewed estimations for the posterior distributions. BDM is the method that shows less skewness.
3. BDM is the method that offers estimations for posterior distribution with less variability. IBDM provides higher variability, but we must keep in mind that this method stresses the importance of extreme values, therefore more variability is expectable than the one provided by BDM. The method with highest variability is MHM.
4. The election of the most suitable method also depends on the characteristics of the problem. When block maxima data are very similar to the baseline distribution, BDM provides the best estimations and the lowest measures of error. On the contrary, when extreme data differ from baseline data, IBDM offers the lowest errors. IBDM is the most stable method: regardless of the differences between extreme data and baseline data, it provides reasonably good measures of error.

### 4.1. Gumbel Baseline Distribution

We considered the baseline $G(\mu_{b},\sigma_{b})$ distribution. As the localization parameter has no effect on the variability of data, its value was fixed as $\mu_{b}=0$ for easiness. Scale parameter does affect variability, so we considered values $\sigma_{b}=2^{j},\;j=-2,-1,0,1,2$.

**Figure 1:**
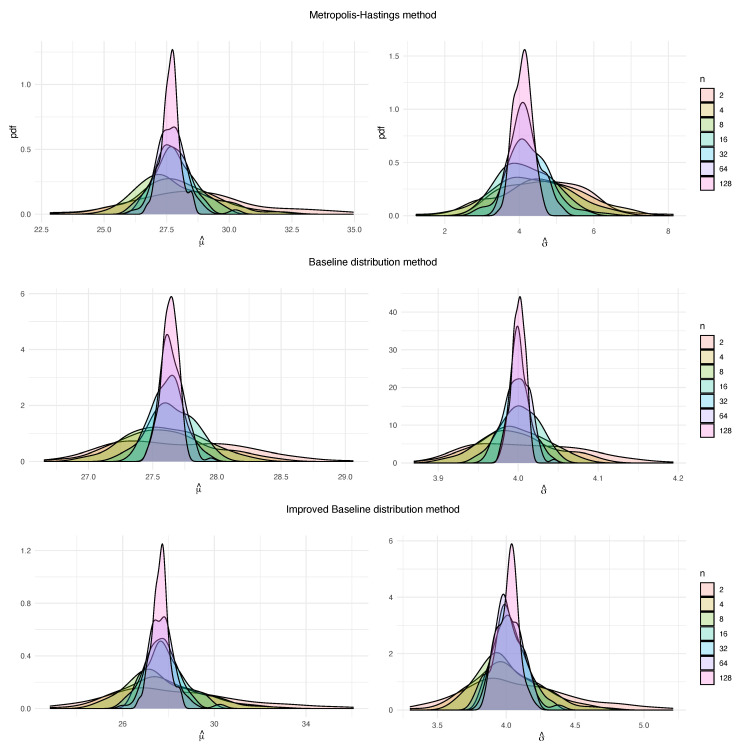
Probability density functions for 100 estimations of the block maxima parameters μ (**left**) and σ (**right**), obtained for the three methods, with k=1000 and different values of *n*, from G(0,4) baseline distribution.

One important point of the simulation study is to observe how the estimation of the parameters vary for a fixed block size as the number of block maxima *n* (the amount of information we have) is changing. Regardless of the chosen method, as *n* increases, variability and skewness decreases. However, for small values of *n*, BDM and IBDM provide more concentrated and less skewed distributions than the ones offered by MHM. We can appreciate this in Figure 1, where the probability density functions (pdf) are shown for the 100 estimations of the mean for the parameters μ (left) and σ (right) for block maxima Gumbel distributions, with the three methods. The baseline distribution is $G\left(0,4\right)$ and fixed block size k=1000. Therefore, block maxima distribution is G(27.63,4) (from (11)). Scales are very different in this charts, due to a better visualization of distributions. For example, for MHM, highest value of the pdf for $\hat{\sigma }$ is around 1.5 (for n=128), but it is over 40 for BDM.

This behavior is shown qualitatively for all the values of the parameters employed in the simulation.

To compute measures of error, in the case of Gumbel baseline distribution, we can employ absolute errors $AE_{i}=\left|{\hat{\theta }}_{i}-\theta \right|$, where ${\hat{\theta }}_{i}$ is the estimation obtained from *i*th sample and θ is the real value of the estimated parameter. We can then define

- Mean error:
  $$\mathrm{ME}=\frac{1}{M}\sum_{i=1}^{M}\left({\hat{\theta }}_{i}-\theta \right).$$
- Root mean square error:
  $$\mathrm{RMSE}=\sqrt{\frac{1}{M}\sum_{i=1}^{M}{\left({\hat{\theta }}_{i}-\theta \right)}^{2}}.$$
- Mean absolute error:
  $$\mathrm{MAE}=\frac{1}{M}\sum_{i=1}^{M}\left|{\hat{\theta }}_{i}-\theta \right|,$$

where M is the number of samples.

The three methods provide estimations with low absolute errors AE when the number of maxima *n* is high, and especially when block size *k* is high. When both numbers are small, BDM and IBDM get closer estimations and differ from MHM.

In Table 2 and Table 3, we show values for ME and RMSE for the estimations of parameters μ and σ, respectively, for some values of *k*, *n* and $\sigma_{b}$, for a $G(0,\sigma_{b})$ baseline distribution. We can see that BDM is the method that offers lower values for both measures of error, followed by IBDM. The method that provides highest errors is MHM.

### 4.2. Exponential Baseline Distribution

Assume now we have another baseline distribution *F*, which is not a Gumbel. Notice that, for methods MHM and IBDM, we are approaching block maxima distribution by a Gumbel. However, for BDM, we employ the *k*th power of the baseline distribution function. When the baseline distribution is a Gumbel, we know that the *k*th power is also a Gumbel. However, for another baseline distributions, this is not true.

For this reason, we have to define different measures of error to evaluate the quality of estimations. We compared estimated distribution functions (*H*) with real ones ($F^{k}$) through their mean distance (D), mean absolute distance (AD) and root square distance (RSD). As analytical computation is not possible, we made a Monte-Carlo computation employing sample size $s=10^{4}$. Then,

(33) $$D_{j}=\frac{1}{s}\sum_{i=1}^{s}\left(H\left(x_{i};{\hat{\theta }}_{j}\right)-F{\left(x_{i};\theta \right)}^{k}\right)$$

(34) $$AD_{j}=\frac{1}{s}\sum_{i=1}^{s}|H\left(x_{i};{\hat{\theta }}_{j}\right)-F{\left(x_{i};\theta \right)}^{k}|$$

and

(35) $$RSD_{j}=\sqrt{\frac{1}{s}\sum_{i=1}^{s}{\left(H\left(x_{i};{\hat{\theta }}_{j}\right)-F{\left(x_{i};\theta \right)}^{k}\right)}^{2}},$$

with j=1,…,M, where M is the number of samples. $H(x;\hat{\theta })$ denotes the estimated distribution function for block maxima, for the baseline parameter $\hat{\theta }$, which is $\hat{\theta }={\hat{\lambda }}_{b}$ if we have an exponential baseline distribution and $\hat{\theta }=\left({\hat{\mu }}_{b},{\hat{\sigma }}_{b}\right)$ for the Normal baseline distribution.

The measures of error were:

- Mean error:
  (36) $$\mathrm{ME}=\frac{1}{M}\sum_{j=1}^{M}D_{j}.$$
- Root mean square error:
  (37) $$\mathrm{RMSE}=\frac{1}{M}\sum_{j=1}^{M}RSD_{j}.$$
- Mean absolute error:
  (38) $$\mathrm{MAE}=\frac{1}{M}\sum_{j=1}^{M}AD_{j}.$$

We considered the baseline $Exp(\lambda_{b})$ distribution. In this case, for *k* big enough, $X\approx G\left(\lambda_{b}^{-1}\ln k,\lambda_{b}^{-1}\right)$. We took $\lambda_{b}=2^{j}$, with j=−2,−1,0,1,2.

**Figure 2:**
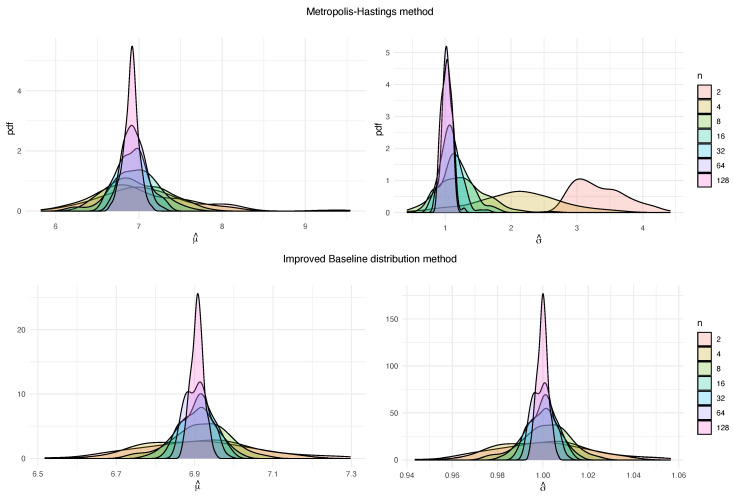
Probability density functions for M estimations of the block maxima parameters μ (left) and σ (right), obtained for the methods MHM and IBDM, with k=1000 and different values of *n*, from Exp(1) baseline distribution.

As in the Gumbel baseline distribution, MHM shows high skewness when the number of blocks *n* is very small, compared to IBDM (see Figure 2). In addition, if we compute measures of error, we can see that, for small block sizes, the three methods offer similar values (see Table 4). For bigger values of *k*, BDM and IBDM provide better results, and usually BDM is the best method.

### 4.3. Normal Baseline Distribution

Finally, assume the baseline distribution is $N\left(\mu_{b},\sigma_{b}\right)$. As the mean has no effect on the variability of data, its value was fixed as $\mu_{b}=0$ for easiness. Standard deviation was taken as $\sigma_{b}=2^{j}$, j=−2,−1,0,1,2.

**Figure 3:**
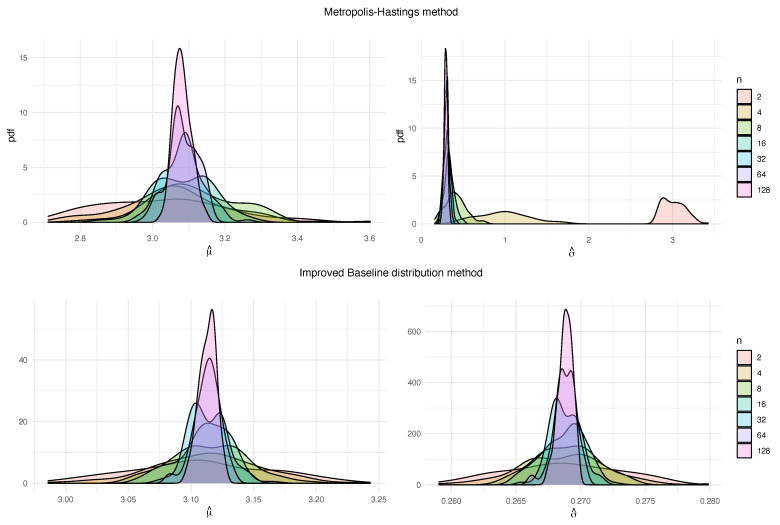
Probability density functions for M estimations of the block maxima parameters μ (left) and σ (right), obtained for the methods MHM and IBDM, with k=1000 and different values of *n*, from N(0,1).

The conclusions we obtain are quite similar to the previous case. We illustrate skewness and variability for MHM employing similar graphs (see Figure 3). Variability is especially pronounced when *n* is small, and we are estimating σ. In addition, errors are shown in Table 5.

In practical situations, data might not adjust to a concrete distribution and some perturbations (noise) could appear. To get a quick overview of how differences between baseline distribution data and block maxima data can affect the choice of the best method, we simulated a simple situation, when data come from a mixture of normal variables. Concretely,

$$Y=0.9\cdot Z+0.1\cdot Y_{1},\;Z∼N\left(0,1\right),\;Y_{1}∼N\left(1,1.5\right).$$

**Figure 4:**
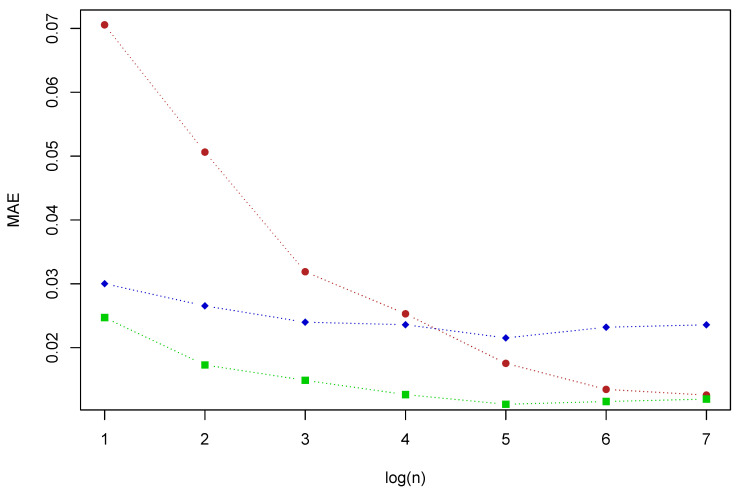
MAE for the three methods MHM (red), BDM (blue) and IBDM (green) with k=100 and different values of *n*, for the baseline distribution *Y*.

In Figure 4, we can see how MAE vary for the three methods when we vary the number of block maxima *n*, for a block size k=100. In this case, IBDM offers the lowest errors, because it stresses the importance of extreme data. When the extreme data are scarce, both new methods, BDM and IBDM, improve MHM meaningfully.

## 5. Conclusions

1. One of the most common problems in EVT is estimating the parameters of the distribution, because the data are usually scarce. In this work, we considered the case when block maxima distribution is a Gumbel, and we developed two bayesian methods, BDM and IBDM, to estimate posterior distribution, making use of all the available data of the baseline distribution, not only the block maxima values.
2. The methods were proposed for three baseline distributions, namely Gumbel, Exponential and Normal, but the new strategy can easily be applied to some other baseline distributions, following the relations shown in Table 1.
3. We performed a broad simulation study to compare BDM and IBDM methods to classical Metropolis–Hastings method (MHM). The results are based on numerical studies, but theoretical support still needs to be provided.
4. We obtained that posterior distributions of BDM and IBDM are more concentrated and less skewed than MHM.
5. In general, the results obtained show that the methods which offer lower measures of error are BDM and IBDM, as they leverage all the data. The classical method, MHM, shows the worst results, especially when extreme data are scarce.
6. IBDM is the most stable method: regardless of the differences between extreme data and baseline data, it provides reasonably good measures of error. When the extreme data are scarce, both new methods, BDM and IBDM, improve MHM meaningfully.

## Figures and Tables

**Table 1:** Normalization constants computed for the most employed distribution function.

Baseline Distribution *F*	$a_{k}$	$b_{k}$
Exponential $\left(\lambda \right)$	$\lambda^{-1}$	$\lambda^{-1}\ln k$
Gamma $\left(\alpha ,\beta \right)$	β	$\beta \left[\ln k+\left(\alpha -1\right)\ln\;\ln k-\ln\Gamma \left(\alpha \right)\right]$
Gumbel $\left(\mu ,\sigma \right)$	σ	μ+σlnk
Log-Normal $\left(\mu ,\sigma \right)$	$a_{N}\;\sigma \;e^{\mu +\sigma \;b_{N}}$	$e^{\mu +\sigma \;b_{N}}$
Normal $\left(\mu ,\sigma \right)$	$\sigma {\left(2\ln k\right)}^{-1/2}$	$\mu +\sigma \left[{\left(2\ln k\right)}^{1/2}-\frac{\ln\ln k+\ln4\pi }{2{\left(2\ln k\right)}^{1/2}}\right]$
Rayleigh $\left(\sigma \right)$	$\sigma \;{\left(2\ln k\right)}^{-1/2}$	$\sigma \;{\left(2\ln k\right)}^{1/2}$

**Table 2:** ME for μ, with RMSE in brackets, for a baseline distribution $G\left(0,\sigma_{b}\right)$.

*k*										
	10			100			1000			
n	MHM	BDM	IBDM	MHM	BDM	IBDM	MHM	BDM	IBDM	$\sigma_{b}$
2	−0.0879 (0.2290)	0.0752 (0.1594)	−0.0038 (0.1848)	−0.1056 (0.2136)	0.0044 (0.0635)	−0.0450 (0.1658)	−0.0495 (0.1659)	0.0061 (0.0343)	0.0159 (0.1782)	1/4
	0.3128 (0.9906)	0.3376 (0.6637)	0.3350 (0.7905)	0.3326 (0.8535)	0.1017 (0.3046)	0.2866 (0.6906)	0.1578 (0.6573)	0.0354 (0.1464)	0.1137 (0.6850)	1
	1.6813 (4.6198)	1.0156 (2.2176)	1.3856 (2.6874)	0.2687 (3.1694)	−0.0001 (1.1367)	0.3466 (2.0640)	1.0252 (2.6346)	0.0747 (0.4899)	0.6297 (2.8024)	4
16	0.0040 (0.0677)	0.0036 (0.0477)	−0.0026 (0.0680)	−0.0018 (0.0645)	−0.0050 (0.0260)	−0.0083 (0.0636)	0.0118 (0.0678)	−0.0002 (0.0119)	0.0014 (0.0636)	1/4
	0.0712 (0.2556)	0.0505 (0.1966)	0.0528 (0.2470)	0.0454 (0.2821)	−0.0033 (0.1019)	0.0218 (0.2657)	0.0499 (0.2536)	0.0006 (0.0441)	0.0219 (0.2446)	1
	0.2096 (1.3466)	0.1711 (0.8447)	0.2450 (1.1989)	0.0625 (1.0148)	−0.0010 (0.3901)	0.0861 (1.0165)	0.2545 (0.8414)	0.0178 (0.1779)	0.2324 (0.8607)	4
128	0.0018 (0.0225)	0.0023 (0.0170)	0.0012 (0.0224)	0.0012 (0.0233)	0.0001 (0.0088)	0.0006 (0.0230)	−0.0038 (0.0208)	−0.0005 (0.0039)	−0.0045 (0.0206)	1/4
	0.0258 (0.0992)	0.0155 (0.0650)	0.0233 (0.0982)	0.0070 (0.0958)	0.0006 (0.0355)	0.0041 (0.0953)	−0.0037 (0.0944)	0.0004 (0.0170)	−0.0067 (0.0948)	1
	−0.0077 (0.3712)	0.0098 (0.2236)	0.0002 (0.3623)	0.0070 (0.3897)	0.0007 (0.1425)	0.0052 (0.3879)	0.0215 (0.3650)	0.0001 (0.0636)	0.0097 (0.3632)	4

**Table 3:** ME for σ, with RMSE in brackets, for a baseline distribution $G\left(0,\sigma_{b}\right)$.

*k*										
	10			100			1000			
n	MHM	BDM	IBDM	MHM	BDM	IBDM	MHM	BDM	IBDM	$\sigma_{b}$
2	2.4739 (2.6201)	0.0309 (0.0584)	0.0258 (0.0560)	2.6628 (2.6967)	0.0013 (0.0119)	0.0048 (0.0216)	2.7177 (2.7371)	0.0007 (0.0045)	0.0024 (0.0155)	1/4
	2.3399 (2.4169) 0.8238 (1.6431)	0.1121 (0.2259) 0.3104 (0.7600)	0.1324 (0.2591) 0.4727 (0.9749)	2.3384 (2.3760) 0.6607 (1.1945)	0.0206 (0.0596) 0.0056 (0.2174)	0.0445 (0.1085) 0.0790 (0.4192)	2.4139 (2.4441) 0.7284 (1.2739)	0.0043 (0.0204) 0.0117 (0.0680)	0.0130 (0.0774) 0.0905 (0.3978)	1
	0.8238 (1.6431)	0.3104 (0.7600)	0.4727 (0.9749)	0.6607 (1.1945)	0.0056 (0.2174)	0.0790 (0.4192)	0.7284 (1.2739)	0.0117 (0.0680)	0.0905 (0.3978)	4
16	0.0292 (0.0577)	0.0006 (0.0165)	0.0031 (0.0279)	0.0229 (0.0560)	−0.0008 (0.0051)	−0.0037 (0.0264)	0.0462 (0.0788)	0.0000 (0.0016)	0.0070 (0.0293)	1/4
	0.1520 (0.2835)	0.0152 (0.0604)	0.0367 (0.1234)	0.1339 (0.2740)	−0.0003 (0.0198)	0.0032 (0.0959)	0.1495 (0.2712)	0.0001 (0.0059)	0.0058 (0.0605)	1
	0.3237 (0.9959)	0.0742 (0.2826)	0.1261 (0.4765)	0.3159 (0.8719)	−0.0010 (0.0755)	0.0176 (0.2238)	0.2071 (0.7576)	0.0023 (0.0237)	0.0294 (0.1211)	4
128	0.0074 (0.0181)	0.0009 (0.0058)	0.0055 (0.0162)	0.0036 (0.0203)	0.0001 (0.0017)	0.0018 (0.0184)	0.0027 (0.0179)	−0.0001 (0.0005)	0.0009 (0.0161)	1/4
	0.0300 (0.0778)	0.0041 (0.0221)	0.0220 (0.0693)	0.0313 (0.0756)	0.0003 (0.0069)	0.0203 (0.0616)	0.0138 (0.0695)	0.0000 (0.0023)	0.0037 (0.0518)	1
	0.0557 (0.2423)	0.0062 (0.0759)	0.0287 (0.1843)	0.0415 (0.2805)	−0.0020 (0.0274)	0.0024 (0.1286)	0.0884 (0.2522)	−0.0001 (0.0086)	0.0123 (0.0753)	4

**Table 4:** ME for σ, with RMSE in brackets, for a baseline distribution $Exp\left(\lambda_{b}\right)$.

*k*										
	10			100			1000			
n	MHM	BDM	IBDM	MHM	BDM	IBDM	MHM	BDM	IBDM	$\lambda_{b}$
2	−0.0645 (0.1569)	0.0287 (0.1297)	0.0207 (0.1278)	−0.0563 (0.1485)	0.0098 (0.0880)	0.0061 (0.0878)	−0.0652 (0.1471)	−0.0004 (0.0339)	−0.0011 (0.0339)	1/2
	−0.0818 (0.2022)	0.0157 (0.1409)	0.0084 (0.1382)	−0.0915 (0.2032)	−0.0078 (0.0780)	−0.0115 (0.0782)	−0.0945 (0.2012)	0.0002 (0.0330)	−0.0006 (0.0330)	1
	−0.0909 (0.2442)	0.0299 (0.1400)	0.0221 (0.1377)	−0.1004 (0.2448)	−0.0033 (0.0673)	−0.0070 (0.0674)	−0.0954 (0.2437)	0.0066 (0.0377)	0.0058 (0.0376)	2
16	−0.0232 (0.0725)	−0.0075 (0.0476)	0.0029 (0.0490)	−0.0263 (0.0743)	−0.0041 (0.0255)	−0.0035 (0.0254)	−0.0224 (0.0705)	−0.0030 (0.0128)	−0.0029 (0.0128)	1/2
	−0.0156 (0.0680)	−0.0006 (0.0440)	0.0096 (0.0452)	−0.0221 (0.0832)	−0.0008 (0.0302)	−0.0002 (0.0302)	−0.0159 (0.0750)	0.0008 (0.0110)	0.0008 (0.0110)	1
	−0.0109 (0.0741)	0.0019 (0.0429)	0.0120 (0.0455)	−0.0230 (0.0750)	−0.0027 (0.0294)	−0.0021 (0.0293)	−0.0220 (0.0743)	0.0001 (0.0132)	0.0122 (0.0132)	2
128	−0.0053 (0.0282)	−0.0050 (0.0197)	0.0076 (0.0229)	−0.0003 (0.0249)	−0.0007 (0.0097)	0.0005 (0.0097)	−0.0093 (0.0251)	−0.0012 (0.0044)	−0.0010 (0.0044)	1/2
	−0.0039 (0.0244)	−0.0057 (0.0183)	0.0069 (0.0218)	−0.0020 (0.0243)	0.0002 (0.0100)	0.0013 (0.0101)	−0.0007 (0.0227)	0.0002 (0.0036)	0.0003 (0.0036)	1
	−0.0010 (0.0243)	−0.0022 (0.0153)	0.0103 (0.0216)	−0.0002 (0.0267)	0.0012 (0.0113)	0.0024 (0.0115)	−0.0004 (0.0269)	0.0012 (0.0044)	0.0013 (0.0044)	2

**Table 5:** ME for σ, with RMSE in brackets, for a baseline distribution $N\left(0,\sigma_{b}\right)$.

*k*										
	10			100			1000			
n	MHM	BDM	IBDM	MHM	BDM	IBDM	MHM	BDM	IBDM	$\sigma_{b}$
2	−0.1097 (0.2923)	−0.2932 (0.3274)	−0.1942 (0.2210)	−0.1120 (0.2975)	−0.1183 (0.1356)	−0.1204 (0.1481)	−0.1101 (0.2991)	−0.0132 (0.0443)	−0.0319 (0.0559)	1/4
	−0.1023 (0.2481)	−0.0049 (0.1139)	−0.0222 (0.1404)	−0.1009 (0.2609)	0.0078 (0.0718)	−0.0077 (0.0876)	−0.1053 (0.2704)	0.0047 (0.0394)	−0.0150 (0.0482)	1
	−0.0822 (0.1643)	0.0252 (0.1197)	−0.0079 (0.1374)	−0.0803 (0.1790)	0.0079 (0.0892)	−0.0103 (0.1073)	−0.0824 (0.1876)	−0.0045 (0.0393)	−0.0227 (0.0497)	4
16	−0.0089 (0.0736)	−0.0650 (0.0765)	−0.0435 (0.0644)	−0.0131 (0.0785)	−0.0166 (0.0326)	−0.0278 (0.0500)	−0.0046 (0.0638)	−0.0006 (0.0134)	−0.0198 (0.0283)	1/4
	−0.0085 (0.0750)	0.0024 (0.0546)	−0.0030 (0.0645)	−0.0092 (0.0658)	−0.0042 (0.0282)	−0.0164 (0.0423)	−0.0109 (0.0724)	0.0012 (0.0144)	−0.0184 (0.0292)	1
	−0.0060 (0.0679)	0.0033 (0.0427)	−0.0049 (0.0574)	−0.0171 (0.0719)	−0.0022 (0.0338)	−0.0185 (0.0472)	−0.0028 (0.0675)	−0.0006 (0.0142)	−0.0194 (0.0281)	4
128	0.0054 (0.0323)	−0.0101 (0.0188)	−0.0116 (0.0299)	0.0041 (0.0282)	−0.0011 (0.0116)	−0.0154 (0.0272)	0.0021 (0.0235)	−0.0008 (0.0044)	−0.0207 (0.0260)	1/4
	0.0090 (0.0336)	−0.0002 (0.0172)	−0.0038 (0.0296)	0.0058 (0.0301)	0.0014 (0.0116)	−0.0126 (0.0263)	0.0016 (0.0249)	−0.0002 (0.0050)	−0.0204 (0.0258)	1
	0.0052 (0.0357)	−0.0020 (0.0188)	−0.0063 (0.0320)	0.0052 (0.0288)	−0.0006 (0.0116)	−0.0147 (0.0272)	0.0020 (0.0277)	0.0001 (0.0048)	−0.0200 (0.0255)	4
